# Obstetrics and Gynecology

**Published:** 1895-04

**Authors:** 


### OBSTETRICS AND GYNECOLOGY.

EDITED BY DR. VIRGIL O. HARDON.

### SYMPHYSEOTOMY IN PRIVATE PRACTICE.

The feasibility of performing symphyseotomy without special apparatus or trained assistants is shown in the report of a case by Wallich (*Annales de Gynécologie et d'Obstetrique*, August, 1894). The patient was thirty-two years of age, V-para, under medium height, and stout. She gave no history of rachitis, nor were there physical signs of such disease. In her first pregnancy she was delivered at term of a living child, weighing 1750 grammes, after twelve hours' labor. The second pregnancy terminated in a miscarriage at two and a half months. In the third pregnancy she was delivered spontaneously of a living child, weighing 2250 grammes, after a labor lasting two and a half hours. Her fourth labor was instrumental, the forceps being applied after forty-eight hours at the superior strait. The child, weighing 3300 grammes, lived. The present labor had existed twenty-four hours when she was seen by Wallich, the dilatation being at that time about the size of a five-franc piece. The membranes then ruptured, and the amniotic liquor was noticed to be greenish in color, the heart above the brim, and showing no tendency to descend.

On the following day the patient was again seen by Wallich, and found very excited, the pains vigorous, and the fetal heart-sounds good. The fetus was presenting by the vertex with Naegele's obliquity. The anterior aspect of the sacrum was accessible, but promontory could not be reached on account of the fetal parts. Three hours later, the dilatation being complete, the patient was placed in the obstetrical position upon a case of drawers. Two physicians, a nurse, and the husband assisted the operator. The patient was anesthetized and forceps applied under antiseptic precautions upon the head still above the pelvic brim. Not securing descent after his first effort, Wallich performed symphyseotomy at once. The cartilage and subpubic ligament were divided while the forceps was in position. The pubic bones separated four centimeters at once, and during traction upon the resisting head separated five and a half centimeters. The head readily descended, was delivered living, and weighed after extraction 3810 grammes. The placenta was next extracted, and an intra-uterine douche administered.

During the delivery of the placenta and the douche the wound had been plugged with iodoform gauze. This was removed and the edges sutured when the pelvis was encircled with a plaster of Paris bandage. During the night the patient was left upon the chest of drawers. Next day she was removed to her bed. Scrupulous cleanliness and antisepsis were practiced, the wound being dressed twice a day, while the external genitals were douched after every micturition and defecation. On the eighth day the deep sutures were removed, and on the following day the superficial ones. The plaster of Paris belt was removed on the thirteenth day. On the twentieth day the patient was able to rise, and walk without pain. The child was suckled from the first, and six weeks after the operation both were flourishing. The sacro-subpubic diameter was found to be 10.7 centimeters. The promontory was very high. The fetal head measurements were as follows: Biparietal, 9.4 centimeters; bitemporal, 18.2; suboccipito-bregmatic, 9.9; suboccipito-frontal, 11.6; occipito-frontal, 12.1; occipito-mental, 14.

The operation was indicated by Naegele's obliquity, the volume of the head, and the contraction of the pelvis. Wallich thinks that peristent attempts at extraction with forceps would have injured the child's skull.

---

### PRINCIPLES OF AXIS TRACTION.

Jewett, Charles (*Brooklyn Medical Journal,* January, 1895), read a paper on this subject before the Brooklyn Gynecological Society. He first stated that while Pajot's maneuver or a similar one overcame in a great measure the defect in the ordinary forceps, yet the exact adjustment of these two forces which it necessitated was impracticable. He considered it a valuable manipulation, but only a rude approximation to axis traction. The aim of the properly constructed axis-traction forceps is to accomplish delivery with the least possible expenditure of force. In order for this to be done, traction must be made absolutely in the line of descent from the beginning to the end of delivery, and the line in which to pull must be made known at every point of descent by a reliable index. The instrument must allow pulling exactly in this line. Once the blades are properly applied, traction should not disturb at any point in the descent the parallelism between the axis of the blades and that region of the canal in which they lie. The handles of the instrument will serve as a constant index to the direction in which the head is moving, and consequently a constant guide to the line of traction. In order for this to be accomplished perfectly, the traction rods should be attached to the blades by a movable joint, and the traction bar to the rods by a universal joint. During the operation the principal point is to keep the traction rods parallel with the handles of the forceps.

The location of the stud from which the traction rods pull is most important. Theoretically, it should be the center of the blades, but since that would fall within the fenestra a point must be selected just below it. According to Dr. Milne Murray, of Edinburgh, this point should fall within a line which is tangent to an arc of a circle described upon a perpendicular line bisecting a line drawn between the center of the tip of the blade and the point on the forceps blade where the pelvic curve begins.

In many axis-traction forceps, including even Tarnier's, the location of the stud is not at the vantage point.

The model of the instrument Dr. Jewett presented was based upon Murray's projection; the pelvic and cranial curve of the Tarnier instrument and the fixation screw were preserved, but the half Smellie was substituted for the button lock. The attachment of the traction bar to the rods is so arranged as to permit less mobility at that point than in most other instruments.

---

### Spurious Hermaphroditism.

The following interesting case was presented before one of the county branches of the British Medical Association:

Mr. Christopher Martin showed a case of spurious hermaphroditism—a person with the external genitals and general characteristics of a woman, but with the essential organs of a man. He was aged twenty-one, had been brought up as a woman, and had earned his living as a nursemaid, kitchenmaid, and barmaid. In February, 1894, he consulted Mr. Martin with reference to a painful swelling in the left inguinal region. Mr. Martin cut down on this and removed a body which proved to be an undoubted testis. The patient's voice was rather low-pitched, but not masculine. There was no trace of beard, whiskers, or mustache. The breasts were flat and poorly developed. There was no hair on the external genitals. These exactly resembled those of a woman, consisting of a labia minora and majora, a vestibule with the meatus urinarius, a small clitoris, and a vaginal ostium, with the remains of the hymen. The vagina was a blind *cul de sac* an inch and a half long, and there was no trace of a uterus. He made a good recovery from the operation. In September a painful lump formed in the right groin, somewhat similar to that which had appeared on the left, and in October, 1894, Mr. Martin cut down on it and removed what proved to be the right testis. He made an excellent recovery. After the first testis was removed hair began to grow on the pubes, and he developed symptoms of hysteria; and after the second was taken away the breasts became swollen and tender and more fully developed. At the same time he complained of "heats and flushes," which recalled those of the menopause. Mr. Martin showed the two testes, and microscopic sections kindly made for him by Professor Allen.—*British Medical Journal.*

---

### A CASE OF PARTUS SEROTINUS.

Resnikow, of Elisabethgard, Russia (*Centralblatt für Gynäkologie*, No. 24, 1894), reports a case of long continuance of pregnancy, which was under his observation, and for whose authenticity he vouches. Patient was a medium-sized, somewhat anemic II-para. The first child was two years old, having been delivered by forceps at the normal termination of pregnancy, on account of a narrow pelvis (conjugata vera, 10–12½ centimeters) and largeness of the product. On January 11, two years later, her menses ceased. On the beginning of February she experienced the symptoms of pregnancy—nausea and vomiting. Resnikow advised her to have labor prematurely produced at the end of the ninth month of pregnancy, on account of the difficulty in her previous labor, but she refused. The labor, reckoned by Naegele's method, should occur on October 18. On December 14, Resnikow again saw the woman, and found her still pregnant. She had experienced from October 18, for six weeks, daily, weak but perceptible pains lasting several hours. These had ceased for the last two weeks. The movements of the child had not been perceived for two days. Previously these had been well felt. On examination it was found that the os was dilated, admitting one finger. The fetal heart-sounds could not be found. Four days later she fell in labor, and after twenty-four hours of intense pains spontaneously gave birth to a male child, considerably macerated and decomposed, eleven months after the beginning of her pregnancy. The placenta was closely adherent, and entire removal with the hand in the uterus failed.— *Univ. Med. Mag.*

